# The effect of using a sports application on the quality of sleep in patients with heart failure: a randomized clinical trial study

**DOI:** 10.1186/s13102-023-00803-3

**Published:** 2024-01-12

**Authors:** Mohsen Arezomand, Mahlagha Dehghan, Zahra Ebrahimi Rigi, Farhad Fatehi, Parvin Mangolian Shahrbabaki

**Affiliations:** 1 Nursing Research Center, Kerman University of Medical Sciences, Kerman, Iran; 2 Department of Nursing, School of Nursing and Midwifery, Iranshahr University of Medical Sciences, Iranshahr, Iran; 3 School of Psychological Sciences, Monash University, Melbourne, Australia; 4 Centre for Online Health, The University of Queensland, Brisbane, Australia

**Keywords:** Heart failure, Quality of sleep, Exercise, Application

## Abstract

**Background:**

Patients with heart failure often suffer from sleep disorders. Due to the side effects of medications used to treat this disorder, non-pharmacological methods may be used to improve these patients’ sleep quality. This study aimed to determine the effect of a sports application on sleep quality in patients with heart failure.

**Methods:**

In this single-blinded randomized clinical trial, 60 patients aged 30–70 referred to Shafa Hospital in Kerman were randomly assigned to control (*n* = 30) and intervention (*n* = 30) groups based on convenience sampling. The intervention group used the sports application for eight consecutive weeks. Data was collected using a demographic questionnaire and the Pittsburgh Sleep Quality Index (PSQI). An independent t-test and a Mann-Whitney U test were used for comparisons between groups and paired t-tests and Wilcoxon tests were used for comparisons within groups.

**Results:**

The data analysis revealed a significant difference in sleep quality scores between the two groups after the intervention, meaning that the intervention group had better sleep quality than the control group (*P* < 0.05).

**Conclusions:**

As a result of the study, it was found that the sport application improved the quality of sleep in patients with heart failure. Our recommendation is for healthcare providers to use this sports application to present educational content related to physical activity and improve the sleep quality of patients.

**Trial registration:**

Iranian Registry of Clinical Trials: IRCT. 2019123045475N1.” Registered 16 December 2019.

## Introduction

As a chronic and progressive cardiovascular disease, heart failure is associated with high morbidity, mortality, and economic costs [1]. Its prevalence increases with aging, and its mortality rate remains high despite medical advances [2]. Approximately one to two% of the world’s population suffers from heart failure [3]. Heart failure with reduced ejection fraction occurs when the left ventricle’s muscle does not pump as efficiently as it should [4], resulting in many problems for the patient, including short sleep times and poor sleeping quality [5].

Patients with heart failure frequently complain about sleep disorders, with approximately 33% suffering from insomnia due to the side effects of medications, mood disorders and stress [5]. Inadequate sleep due to the increased release of catecholamines, high blood pressure and oxygen required by the heart increases the cardiac workload and significantly impacts the daily activities and physical and mental health of patients with heart failure [6].

Regular physical activity is a simple and inexpensive method to improve sleep quality [7], in patients with heart failure because it improves cardiac function, reduces cardiac symptoms and sleep latency, and increases sleep duration [8]. However, most patients with heart failure do not participate in prolonged and regular physical activity [9]. Studies have shown that patients with heart failure have insufficient knowledge of self-care measures at home and believe they should not engage in physical activity [9, 10]. Mobile learning provides services to patients without direct supervision from healthcare personnel, which enhances the quality of life and reduces the number of hospitalizations and costs [11], so it provides counselling and educational services to patients [12].

Few studies evaluated sleep disorders from the perspectives of patients with heart failure [13]. Studies suggested that physical activity increased sleep quality in patients undergoing hemodialysis, those with diabetes, cancer and rheumatoid arthritis [8]. However, the evidence shows that patients with heart failure do less physical activity than healthy people due to insufficient awareness of self-care measures [9, 10]. A descriptive study investigated the relationship between physical activity and the sleep quality of patients with heart failure, but it could not evaluate the sleep quality over time and only reflected the patients’ perception of sleep quality [8]. Therefore, it is essential to investigate the effect of using a sports application on the quality of sleep of patients with heart failure.

Despite mobile learning’s ease of use without direct supervision, it seems no one has considered its effect on improving the quality of sleep in heart failure patients and on doing physical activity at home. In addition, the cultures and customs of societies affect mobile learning, physical activity and sleep habits, which have not been studied in Iran. Therefore, this study aimed to investigate the effect of using a sports application on sleep quality in patients with heart failure.

## Objective

This study compared using a sports application to improve sleep quality in heart failure patients with drug therapy as the usual treatment. The best sleep quality is achieved by sleeping more in bed (at least 85% of the time), falling asleep in 30 min or less, waking up less frequently, and staying awake no longer than 20 min after waking up. We hypothesized that the intervention of sports exercises using a sports application would achieve this objective. Therefore, the study’s primary outcome was the prevalence of clinically significant improved sleep quality after eight weeks in heart failure patients.

## Method

### Trial design, participants and setting

This clinical trial is a randomized, pre-posttest, single blind, control group design was conducted on patients with heart failure. Participants met the following criteria: 30–70 years old with class II or class III heart failure, less than 40% ejection fraction, having been diagnosed with heart failure for at least three months, without neurological conditions, vision or hearing difficulties, or severe mental illness, and authorized by their physician to exercise. Patients who did not complete the exercises provided by the application for more than three sessions (one week) or answered fewer than one-third of the questionnaire questions were excluded from the study. This study recruited participants from the Cardiac Rehabilitation Center at Shafa Hospital, an affiliate of Kerman University of Medical Sciences. Patients suffering from joint disease, undergoing cardiac surgery, and having a pacemaker, as well as those with renal failure, received healthcare services at the Cardiac Rehabilitation Center.

### Outcomes and measurements

In this study, sleep disturbance was the primary outcome. Researchers also, used sports applications to improve the sleep quality of heart failure patients.

This study evaluated the demographic characteristics and sleep quality of patients. The demographic questionnaire and the Pittsburgh Sleep Quality Index (PSQI) were used to collect data. The demographic characteristics included age, gender, marital status, type of illness, level of education, place of residence, underlying illness, addiction, occupation, medication regimen, and sleep disorders. Basic information was obtained through personal interviews with patients, their family members or other caregivers and their motor ability, vision and hearing impairment were assessed through physical examination.

The PSQI was used to measure the sleep quality of patients in the last month. It included 19 items with seven subscales: sleep duration, sleep disturbance, sleep latency, daytime dysfunction due to sleepiness, sleep efficiency, overall sleep quality, and sleep medication use. PSQI used a 4-point Likert scale ranging from 0 = no difficulty and 3 = severe difficulty and generated scores according to each domain of the scale. Totaling the averages of the seven factors yields a PSQI score ranging from 0 to 21, with 0–4 indicating “good” sleep and 5–21 indicating “poor,” with the higher total score indicating poorer sleep. As per the authors, a score > 5 indicates a significant disturbance in sleep [14]. The validity and reliability of the questionnaire have been confirmed in the Iranian population [15]. This tool has been used in many studies to measure sleep quality [16, 17]. CONSORT checklist was used to report the research.

### Sample size and sampling

Based on a previous study examining the effect of walking at home on improving the quality of life of patients with heart failure, the dropout rate and test power was 80%; the present study considered a sample size of 60 (with 30 participants in each group) for comparing sleep quality between two groups [18]. Following coordination with the hospital and cardiac rehabilitation center heads, the researchers selected eligible patients. The samples were selected using convenience sampling method, and they were allocated into 2 groups by block randomization method. Labels A and B (A = intervention, B = control) were assigned to the groups, and the block size was 4. The randomization list was generated by using free online software (https://www.sealedenvelope.com/simple-randomiser/v1/lists). MD generated the randomization list and MA rzoomand enrolled the participants and assigned them to the two groups.

**Fig. 1:**
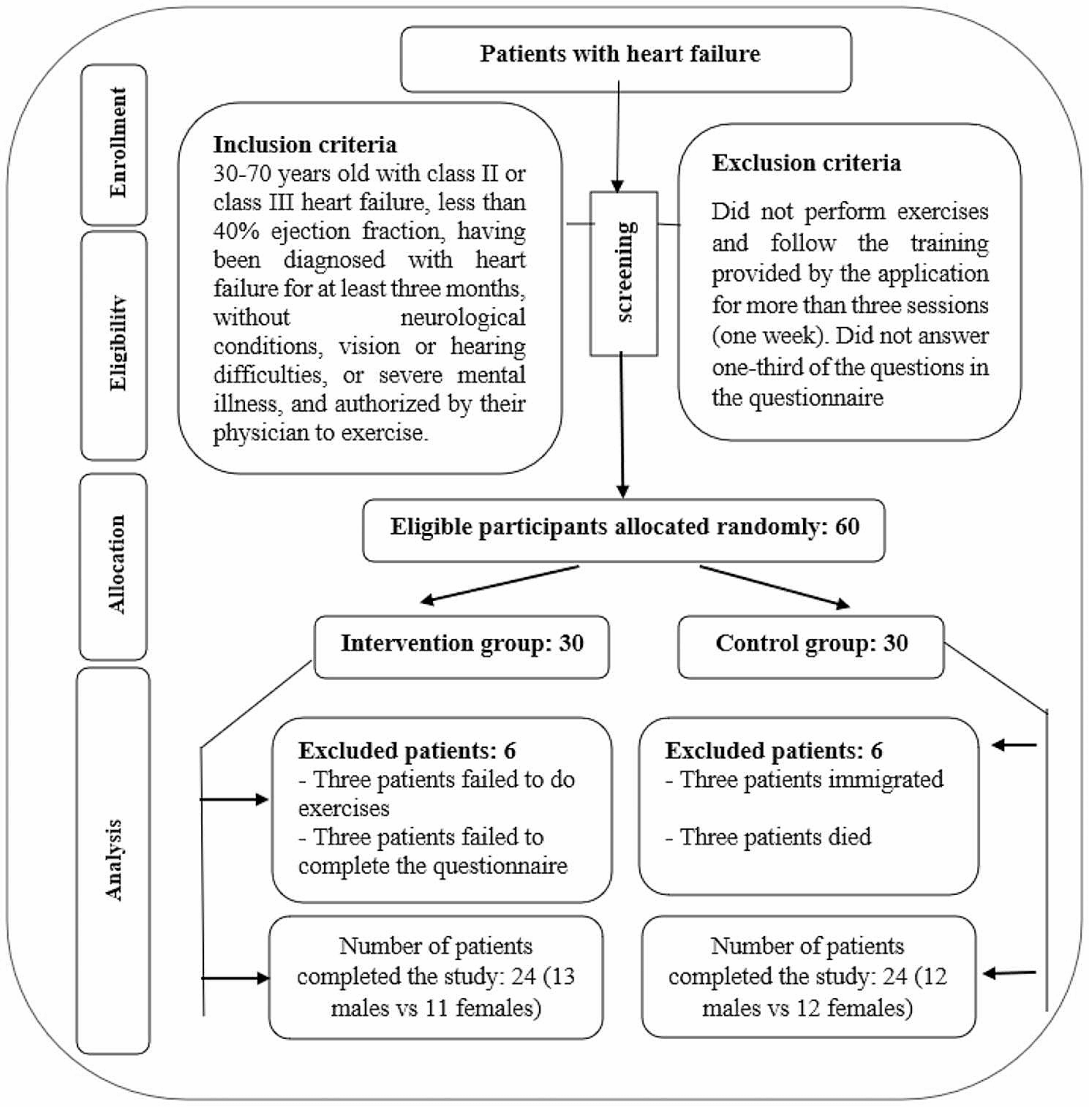
Explanation of sample size and sampling

At first, all the patients with heart failure referred to Shafa Hospital in Kerman who had met the inclusion criteria and were willing to participate were regarded as the research sample. Then, the necessary explanations about the research procedure were given to the samples, and written informed consent was obtained. Based on random block allocation, the eligible patients were divided into two groups. As a result of this method, the number of people assigned to each group is usually almost equal. From 60 eligible patients, 48 (24 in each group) completed the study. The two main reasons for dropouts were: the inability to exercise because of chronic fatigue caused by the disease and failure to answer more than one-third of the questionnaire questions (Fig. 1). The present study is a single-blind randomized clinical trial. Participants was blinded after assignment to interventions.

### Interventions

At baseline, a cardiologist examined participants to ensure they had no medical restrictions. Personal interviews and medical records were used to collect demographic data. The researcher collected data on the sleep quality pattern before and eight weeks after the intervention in both group by the Pittsburgh Sleep Quality Index. One week after the patients’ enrollment, the intervention was conducted. The authors coordinated and monitored their exercise adherence by calling them on a predetermined schedule. Immediately after baseline data collection, randomized participants to the intervention group participated in the sports application intervention. For eight consecutive weeks, participants exercised three times per week. Also, participants were prohibited from improving or disturbing their sleep quality or reading materials related to sleep. In order to design and develop the application, the researchers met with a cardiologist and two medical informatics experts. In addition, ten faculty members approved the educational content before the study. Several stages of the review were conducted during the development of the application for a year. A team of experienced IT engineers, programmers and graphic designers worked together to develop the application. The application was finally approved in August 2021. The application was piloted with ten patients before the study, and any potential problems were resolved. The researcher trained patients and their families in the use of the application prior to the study under the supervision of a cardiologist. The authors provided an explanation of how to use the application. This sports application included texts, podcasts, and educational videos regarding sports movements and conditions for starting and stopping sports. There were two parts to this application providing recommendations before engaging in sports and sports exercises, which will be discussed in the following paragraphs. Before each session at home, it is important to observe the following points: conducting sports in a balanced climate (not too cold or too hot) is important, exercise as desired (in the morning after breakfast or in the evening after dinner), When performing sports in public places, it is advisable to wear a mask when exercising, it is advisable to carry sweet food with you, do not continue exercising if you are experiencing severe shortness of breath, an abnormally high heart rate, excessive fatigue, or chest pain. Sports exercises: the warm-up exercise was made as an animation and included five sports movements in a two-minute educational clip. The walking exercise consists of 30 min of aerobic walking based on the patient’s physiological capabilities. After the patients had been able to perform the exercises for 15 min for two consecutive weeks, they were added 5 min to their training every two weeks until a maximum of 30 min was reached at the end. The cool-down exercise included four soft movements presented as educational clips (animation), as summarized in Table 1. To monitor adherence to exercise, the researcher called each participant three times a week on predetermined days. Depending on the patient’s educational needs and questions, the duration of the phone call varied. In total, he made 24 telephone calls to each patient. Control group participants received drug therapy for sleep disorders, which is the usual treatment. This study lasted five months, from August 2021 to January 2022.

**Table 1:** Summary of exercises provided to patients by the sports application

1	Warm up	Five physical activities: - Jogging for one minute - Walking for one minute, including running in place (20–30 times), raising the hands and legs up and down (20–30 times) and stepping forward and backward (20–30 times) - Raising hands in the air three to four times for one minute - Rotating shoulders: ten times for one minute - Bending the knees for one minute
2	Walking	− 30-minute walking based on the patient’s physiological ability (five minutes were added to the duration of their walking every two weeks up to a maximum of 30 min in the last sessions) - The first and second weeks: 15 min - The third and fourth weeks: 20 min - The fifth and sixth weeks: 25 min - The 7th and 8th weeks: 30 min
3	Cool-down	- Four movements to cool down after 5-minute walking - Holding the ankle while standing - Stretching the trunk from both sides - Performing quad stretch - Performing thigh and hip stretch

### Statistical methods

We analyzed the data using descriptive and inferential statistics by SPSS 25. In order to describe participants’ characteristics and other variables, descriptive statistics (frequency, percentage, mean, and standard deviation) were used. Participants’ characteristics in the intervention and control groups were compared using independent t-tests, chi-squared tests, and Fisher’s exact tests. Considering the normal distribution and Levene’s Test of Equality of Error Variances were fulfilled, Analysis of covariance (ANCOVA) was used to compare the sleep quality score between the two groups after the intervention. In addition, paired t-tests were used for within-group comparisons. This research considered a 95% confidence level, *p*-value = 0.5.

## Results

The study results showed that the mean ages of the intervention and control groups were 49.71 ± 8.73 and 55.21 ± 87.73, respectively. Additionally, we measure clinical data about patients (Table 2). We found no significant differences in age, gender, duration of illness, marital status, occupation, place of residence, hypertension, hyperlipidemia, or history of addiction between the two groups (*P* > 0.05). However, there were significant differences in education level and history of diabetes (*P* < 0.05) (Table 3).

**Table 2:** Comparison of patients’ characteristics between the intervention and control groups

Group		The intervention group		The control group		Statistical test^*^
Variable		Mean	SD	Mean	SD	
Age		49.71	8.73	55.21	10.87	-1.93^a^
Diseas duration (month)		29. 9	11.54	9.75	4.49	-0.18 ^a^
		**n**	**%**	**n**	**%**	
Sex	Female	11	45.8	12	50	0.08^b^
	Male	13	54.2	12	50	
Marital status	Married	23	95.8	22	91.7	0.36^b^
	Single, widowed, divorced	1	4.2	2	8.3	
Education level	Uneducated	5	20.8	12	50	9.11 ^b^
	Middle/high school	6	25	8	33.3	
	Diploma	5	20.8	3	12.5	
	Academic	8	33.3	1	4.2	
Job	Unemployed/housewife	7	29.2	12	50	4.36 ^b^
	Employed	7	29.2	2	8.3	
	Self-employed	7	29.2	8	33.3	
	Retired	3	12.4	2	8.4	
Place of residence	City	20	83.3	18	75	0.58 ^b^
	Village	4	16.7	6	25	

a Independent T Test

b Chi-squared Test

*P < 0.05

**Table 3:** Comparison of patients’ clinical information between the intervention and control groups

Group		The intervention group		The control group		Statistical test	*P*-value
Variable		n	%	n	%		
History of hypertention	Yes	19	79.2	17	70.8	0.44 ^b^	0.5
	No	5	20.8	7	29.2		
History of hyperlipidemia	Yes	10	41.7	6	25.0	1.50^b^	0.22
	No	14	58.3	18	75.0		
History of diabets	Yes	2	8.3	8	33.3	4.55 ^b^	0.03
	No	22	91.7	16	66.7		
History of addiction	Yes	4	20.8	10	41.7	3.63 ^b^	0.06
	No	20	79.2	14	58.3		

b Chi-squared Test

The study results showed that the mean sleep quality scores in the control and intervention groups were 9.42 ± 3.93 and 9.54 ± 3.44, respectively, before the intervention (*P* = 0.91). At the same time, we found a significant difference in the sleep quality scores between the two groups after the intervention, meaning that the sleep quality score in the intervention group improved significantly compared with the control group (*P* < 0.001) (Table 4). The mean scores of various subscales of the PSQI were significantly different between the intervention and control groups after the intervention, except for sleep duration, sleep efficiency and use of sleep medications (*P* < 0.001). Within-group comparison indicated a significant difference in the scores of different subscales of the PSQI in the intervention group before and after the intervention, except for sleep efficiency and use of sleep medications (*P* = 0.001). Sleep duration and latency scores were poor in the control group (*P* = 0.15).

**Table 4:** Comparison of the sleep quality scores between the intervention and the control before and after intervention

Time Group	Sleep quality (Before intervention)		Sleep quality (After intervention)		Mean Difference		Paired t test (*P* value)	Effect size
	Mean	SD	Mean	SD	Mean	SD		
Intervention group	9.54	3.44	6.54	1.93	-3.00	3.09	-4.75 (*P* < 0.001)	1.23
Control group	9.42	3.93	10.08	3.46	0.67	2.18	1.50 (*P* = 0.15)	0.31
Source*	**Type III Sum of Squares**		**df**	**Mean Square**		**F**	***P*** **value**	**Effect size**
Group	78.01		1	78.01		18.66	< 0.001	0.37
Sleep quality Before intervention	227.98		14	16.28		3.89	0.001	0.63

*Analysis of covariance

## Discussion

The present study showed the effect of this sports application on the quality of sleep of the patients with heart failure in the intervention group, but the sleep quality and most of its subscales were significantly lower in the control group. This study could change patients’ views on physical activities as a non-pharmacological method and help healthcare providers to provide counselling and educational services to patients through mobile phones.

The study results indicated that most of the participants in both groups had poor quality of sleep before the intervention. Previous studies found that heart failure patients have poor sleep quality [19, 20]. Sleep deprivation increases heart burden by increasing catecholamine production, blood pressure, and oxygen; consequently, it leads to physical and mental problems and may affect heart failure symptoms, including sleep disorders [21]. Additionally, sleep quality is important for promoting health, and there is a strong connection between sleep disorders and cardiovascular diseases, cancer, and depression [22]. Because of heart failure and its complications, the patients’ quality of sleep was significantly reduced, and sleep disorders could negatively impact their health.

So, these patients had to take sleep medications [23, 24]. Considering the number of drugs used by these patients, it becomes necessary to use non-medicinal methods to improve their sleep quality [8]. However, some studies suggested that patients with heart failure had good sleep quality and needed no sleep medications [25, 26]. Various factors affected the results of the studies, including the severity of the disease, self-care training after discharge, the availability of supporting resources, the lifestyle of the patients, and their demographic and cultural characteristics. Therefore, changing the lifestyle and improving self-care methods can be effective in improving the sleep quality of these patients.

There was a significant difference in sleep quality between the control and intervention groups, with the quality of sleep in the control group deteriorating over time. We concluded that exercising improved sleep quality for patients with heart failure. In line with our findings, Esnaasharieh et al. (2022) found that patients who exercised more experienced better sleep quality [8]. Another study found that aerobic and resistance exercises improved the quality of sleep of older adults with heart failure [27]. Additionally, Hasani Sadi et al. (2016) demonstrated that exercise improves sleep quality, heart function, and motivation [28]. According to research, exercise increases energy expenditure, improves neuropsychological function, and enhances sleep quality [22]. Although many studies have focused on the role of education in improving the sleep quality of patients with heart failure [23], and exercise has been shown to improve sleep quality [8, 29], the majority of patients with heart failure do not engage in physical activity on a regular and prolonged basis [9]. As a result, verbal education and written educational materials are essential to care measures during hospitalization and discharge, but they are insufficient and must be permanent [30]. Due to the increasing use of mobile phones, we developed an application to deliver educational content to patients, as mobile learning reduces hospitalization costs, improves self-care performance, and enhances the patient’s quality of life [12]. Therefore, healthcare providers should utilize this technology to train non-pharmacological methods and achieve better results.

This study had some limitations: the patients’ knowledge and previous experiences influenced their motivation and desire. Moreover, patients were reluctant to exercise because of chronic fatigue, but the researcher encouraged them to balance physical activity with rest periods. Another limitation of the present study was the small number of samples. Since the present study was conducted during the COVID-19 pandemic, the availability of samples was limited. The application development was time-consuming. Furthermore, we recommend that future studies measure sleep quality using objective instruments.

## Conclusions

According to the study results, the use of sports application improved the quality of sleep in patients with heart failure. Since these patients use many drugs during their illness, non-pharmacological methods can play an effective role in their lives. As the modern life is accompanied by the advancement of technology, it is necessary to integrate technology into therapeutic approaches. Therefore, we suggest using this type of treatment to improve the health of patients more than before, but we require more interventions to identify its effectiveness and resolve its defects.

## Data Availability

The datasets used and/or analyzed during the current study are available from the corresponding author on reasonable request.
